# Supplementary material for: NIAPU: network-informed adaptive positive-unlabeled learning for disease gene identification
Source: Bioinformatics. 2023 Feb 2;39(2):btac848. doi: 10.1093/bioinformatics/btac848 (PMC9933847; doi:10.1093/bioinformatics/btac848)
Supplement: btac848_Supplementary_Data [file btac848_supplementary_data.zip › Supplementary File 2.pdf]

Supplementary Material (File 2) to "NIAPU: Network-Informed Adaptive Positive-Unlabeled learning for disease gene identification"

Paola Stolfi, Andrea Mastopietro, Giuseppe Pasculli, Paolo Tieri and Davide Vergni

1 List of supplementary files

- Supplementary File 1: PPI largest connected component used in the experiments;
- Supplementary File 2: this file;
- Supplementary File 3: TFO features.

Computed NeDBIT features can be found in the GitHub repository (`https://github.com/AndMastro/NIAPU`) and details on the PUDI features in the original publication.

2 Data processing details

We checked the presence of each gene from GDAs in the BioGRID dataset and the we removed the absent ones. GDAs derived from DisGeNET comes with a score that defines the relevance of each gene for the disease. These scores range from 0 to 1, take into account the number and type of sources (level of curation, model organisms), and the number of publications supporting the association[1]. Gene associations for the diseases of interest can be found in the GitHub repository.

3 Extended DisGeNET dataset details

The number of associated genes in the extended dataset is 6114 for disease C0006142, 4745 for C0009402, 3974 for C0376358, 2545 for C0036341, 2037 for C3714756, 1583 for C0011581, 1058 for C0005586, 836 for C0023893, 535 for C0001973 and 449 for C0860207.

4 TFO features description

The interactome was analyzed to recover standard centrality measures as degree, degree centrality, betweenness centrality, eigenvector centrality, clustering coefficient, closeness centrality, and current closeness using the Python [1] `NetworkX` graph analysis package [2]. The study of the

[1] `https://www.disgenet.org/dbinfo`

topological structure of PPI networks and of functional/ontological annotations (e.g., centrality measures and gene ontology classification) can provide valuable information on the relevance of a particular gene for a specific biological and pathological process [3, 4]. These metrics have been used as a first part of gene feature vectors. In addition, as a second part of the feature vector, Gene Ontology (GO) information related to the biological processes annotation class of each gene and its first neighbors in the interactome has been retrieved. The Gene Ontology knowledgebase [5] was used for this task. This part of the vector consists in 60 fields; 30 fields in binary format stating if a gene is associated with the given GO term (1) or not (0) and 30 fields indicating the number of neighbors of a gene associated with the given GO term. For any gene, the terms considered were: GO:0008150, GO:0007610, GO:0022610, GO:0044848, GO:0065007, GO:0110148, GO:0009758, GO:0009987, GO:0098754, GO:0032502, GO:0040007, GO:0002376, GO:0044419, GO:0051703, GO:0051179, GO:0040011, GO:0008152, GO:0051704, GO:0032501, GO:0048519, GO:0019740, UBERON:0000062, GO:0043473, GO:0048518, GO:0050789, GO:0000003, GO:0022414, GO:0050896, GO:0048511, GO:0023052.

5 Considerations on NetRing feature

The concept of ring leads to the introduction of a ranking between nodes, i.e., ring zero includes all seed nodes, ring one includes all the nodes that are directly connected to at least one seed node, and so on as the ring level grows. But, when dealing with seed nodes representing disease genes, it is also evident that not all the nodes in each ring are equivalent, for example, there may be a node in the first ring that has only one direct contact with a seed node while another node in the first ring may be directly connected to many seed nodes (it is to be noted that this concept resembles and extends connectivity significance [6]). So, to rank nodes belonging to the same ring, it is important to consider the number of nodes on the lower ring a node is connected to, together with their ranks.

6 ML methods details

We recall we used three ML methods for classification, namely MLP, RF and SVM. We used a MLP with two fully-connected hidden layers (64 and 32 neurons) with ReLU activation function, a dropout layer with dropout probability of 0.3 to prevent overfitting, batch size of 32, Adam [7] optimizer with learning rate of $1e{-}4$. Cross entropy was used as loss function and the network was trained for a maximum of 100 epochs, early stopping when no further improvements in the loss are noticed. The network was developed relying on `Tensorflow 2` [8] and `Keras` [9]. For the SVM, a RBF kernel was used. As for the RF, we employed 100 trees. The rest of the hyperparameters for both SVM and RF are the `Scikit-learn` implementation-default ones [10]. We did not put much emphasis on model fine-tuning since it was not the main goal of our work. Widely used hyperparameters proved to be effective for our classification needs to verify the efficacy of the NIAPU framework.

7 Classification performances

In this section we present the average classification performances for the 5-fold cross-validation on the diseases under study. We recall that the cross-validation was performed on the 70% of the dataset. The features were properly scaled using `Scikit-learn` RobustScaler [10] before being fed to the ML algorithms. By inspecting the tables in Section 7.1 we notice the effectiveness of the synergy between NeDBIT features and APU in properly separating the classes. We also show the results in terms of confusion matrices on the test set in and Section 7.2 (results for malignant neoplasm of breast can be found in the main paper). NeDBIT features allow for a coherent identification of the P class, unlikely TFO and PUDI features. Moreover, we notice that APU is able to perform a meaningful label propagation, despite the features used. However, the P class is identified only by using NeDBIT features, as explained. The three ML methods show different performances. Considering NeDBIT features, RF is the most accurate one on all the classes, MLP delivers a slightly lower accuracy on the LN class. However, most of the wrongly predicted LN samples are misclassified as RN, which is still an acceptable error which could be mitigated by choosing a different labeling strategy from the APU ranking (e.g. increasing the number of RN samples by selecting different quantile thresholds). Finally, SVM shows the lower accuracy, classifying LP samples as WN and RN or the latter as LN (however, still in the negative spectrum). For some diseases, also the LP samples are not always properly classified, proving SVM to be the less effective ML method in this scenario.

7.1 Cross-validation performances using NeDBIT, PUDI and TFO features

The following tables show the cross-validation performances using the different feature sets (Table 1 for TFO, Table 2 for PUDI and Table 3 for NeDBIT) as pooled mean and standard deviation. We notice the incapability of TFO and PUDI features to distinguish the positive samples in class P (see the recall score of this class); this can be achieved only by using NeDBIT features.

7.2 Performances on the test set

We show here in figs. 1 to 9 the performances on the test set comparing the usage of TFO, PUDI and NeDBIT features. We obtained result analogous to the ones seen in the cross-validation.

Table 1: Classification scores as pooled mean and standard deviation (over all diseases) for the 5-fold cross-validation using TFO features.

label	precision	recall	F1 score
**MLP**			
P	0.229 ± 0.238	0.014 ± 0.013	0.026 ± 0.024
LP	0.853 ± 0.014	0.895 ± 0.017	0.873 ± 0.009
WN	0.861 ± 0.015	0.863 ± 0.016	0.861 ± 0.009
LN	0.929 ± 0.01	0.962 ± 0.009	0.945 ± 0.006
RN	0.928 ± 0.011	0.951 ± 0.013	0.939 ± 0.008
macro avg	0.76 ± 0.048	0.737 ± 0.005	0.729 ± 0.006
weighted avg	0.872 ± 0.011	0.89 ± 0.007	0.878 ± 0.008
accuracy	0.89 ± 0.007		
**RF**			
P	0.136 ± 0.233	0.003 ± 0.005	0.006 ± 0.009
LP	0.916 ± 0.011	0.945 ± 0.008	0.93 ± 0.007
WN	0.898 ± 0.009	0.923 ± 0.01	0.91 ± 0.008
LN	0.943 ± 0.008	0.971 ± 0.006	0.956 ± 0.005
RN	0.965 ± 0.007	0.986 ± 0.006	0.976 ± 0.005
macro avg	0.771 ± 0.047	0.766 ± 0.003	0.756 ± 0.003
weighted avg	0.907 ± 0.01	0.928 ± 0.005	0.914 ± 0.006
accuracy	0.928 ± 0.005		
**SVM**			
P	0.04 ± 0.2	0.001 ± 0.003	0.001 ± 0.006
LP	0.773 ± 0.019	0.834 ± 0.013	0.799 ± 0.012
WN	0.783 ± 0.015	0.795 ± 0.016	0.784 ± 0.012
LN	0.906 ± 0.013	0.917 ± 0.011	0.912 ± 0.01
RN	0.885 ± 0.017	0.881 ± 0.024	0.881 ± 0.017
macro avg	0.677 ± 0.04	0.685 ± 0.008	0.675 ± 0.008
weighted avg	0.812 ± 0.012	0.831 ± 0.01	0.818 ± 0.011
accuracy	0.831 ± 0.01		

Table 2: Classification scores as pooled mean and standard deviation (over all diseases) for the 5-fold cross-validation using PUDI features.

label	precision	recall	F1 score
**MLP**			
P	0.236 ± 0.245	0.022 ± 0.015	0.038 ± 0.026
LP	0.82 ± 0.023	0.878 ± 0.019	0.847 ± 0.015
WN	0.772 ± 0.019	0.787 ± 0.028	0.778 ± 0.017
LN	0.828 ± 0.021	0.858 ± 0.016	0.842 ± 0.014
RN	0.912 ± 0.02	0.893 ± 0.028	0.902 ± 0.017
macro avg	0.714 ± 0.05	0.687 ± 0.01	0.682 ± 0.011
weighted avg	0.817 ± 0.013	0.831 ± 0.011	0.82 ± 0.012
accuracy	0.831 ± 0.011		
**RF**			
P	0.209 ± 0.163	0.025 ± 0.019	0.044 ± 0.032
LP	0.687 ± 0.017	0.741 ± 0.016	0.711 ± 0.013
WN	0.593 ± 0.02	0.609 ± 0.02	0.6 ± 0.015
LN	0.647 ± 0.022	0.691 ± 0.019	0.668 ± 0.015
RN	0.801 ± 0.018	0.746 ± 0.018	0.772 ± 0.012
macro avg	0.587 ± 0.034	0.563 ± 0.008	0.559 ± 0.009
weighted avg	0.67 ± 0.01	0.678 ± 0.009	0.671 ± 0.009
accuracy	0.678 ± 0.009		
**SVM**			
P	0.03 ± 0.141	0.0 ± 0.001	0.0 ± 0.002
LP	0.618 ± 0.019	0.678 ± 0.028	0.641 ± 0.017
WN	0.504 ± 0.02	0.594 ± 0.021	0.543 ± 0.013
LN	0.6 ± 0.025	0.523 ± 0.024	0.557 ± 0.014
RN	0.67 ± 0.024	0.605 ± 0.021	0.626 ± 0.016
macro avg	0.484 ± 0.029	0.48 ± 0.008	0.474 ± 0.008
weighted avg	0.584 ± 0.012	0.583 ± 0.01	0.576 ± 0.01
accuracy	0.583 ± 0.01		

Table 3: Classification scores as pooled mean and standard deviation (over all diseases) for the 5-fold cross-validation using NeDBIT features.

label	precision	recall	F1 score
**MLP**			
P	0.992 ± 0.011	0.998 ± 0.007	0.995 ± 0.006
LP	0.972 ± 0.008	0.97 ± 0.009	0.971 ± 0.006
WN	0.96 ± 0.011	0.939 ± 0.01	0.948 ± 0.008
LN	0.857 ± 0.028	0.751 ± 0.026	0.792 ± 0.017
RN	0.739 ± 0.025	0.851 ± 0.054	0.781 ± 0.036
macro avg	0.904 ± 0.005	0.902 ± 0.009	0.898 ± 0.01
weighted avg	0.893 ± 0.007	0.883 ± 0.009	0.882 ± 0.011
accuracy	0.883 ± 0.009		
**RF**			
P	1.0 ± 0.0	1.0 ± 0.0	1.0 ± 0.0
LP	0.981 ± 0.006	0.98 ± 0.006	0.981 ± 0.004
WN	0.972 ± 0.007	0.973 ± 0.006	0.972 ± 0.005
LN	0.983 ± 0.006	0.984 ± 0.005	0.984 ± 0.004
RN	0.991 ± 0.004	0.988 ± 0.006	0.99 ± 0.003
macro avg	0.986 ± 0.002	0.985 ± 0.002	0.985 ± 0.002
weighted avg	0.982 ± 0.003	0.982 ± 0.003	0.982 ± 0.003
accuracy	0.982 ± 0.003		
**SVM**			
P	0.996 ± 0.008	1.0 ± 0.0	0.998 ± 0.004
LP	0.867 ± 0.019	0.694 ± 0.021	0.767 ± 0.015
WN	0.618 ± 0.018	0.838 ± 0.016	0.698 ± 0.013
LN	0.666 ± 0.283	0.482 ± 0.019	0.378 ± 0.029
RN	0.398 ± 0.016	0.597 ± 0.008	0.476 ± 0.011
macro avg	0.709 ± 0.059	0.722 ± 0.005	0.663 ± 0.009
weighted avg	0.661 ± 0.074	0.666 ± 0.011	0.598 ± 0.015
accuracy	0.666 ± 0.011		

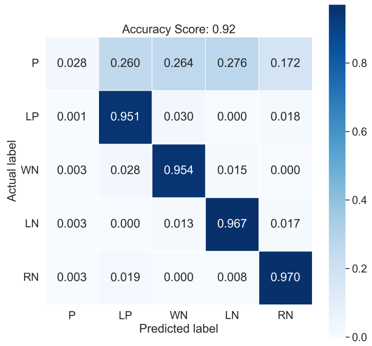

(a) MLP + TFO features

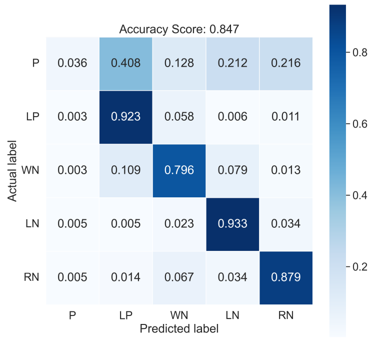

(b) MLP + PUDI features

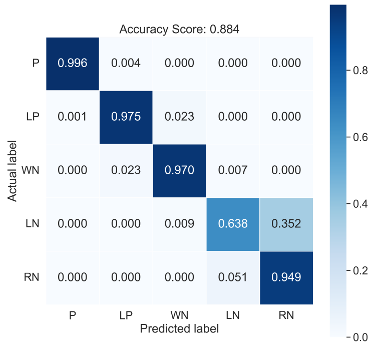

(c) MLP + NeDBIT features

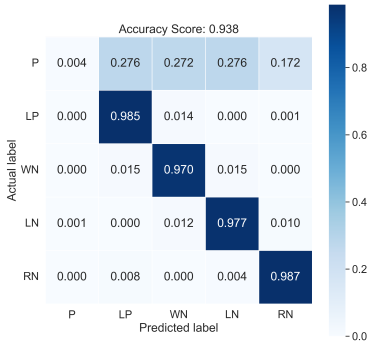

(d) RF + TFO features

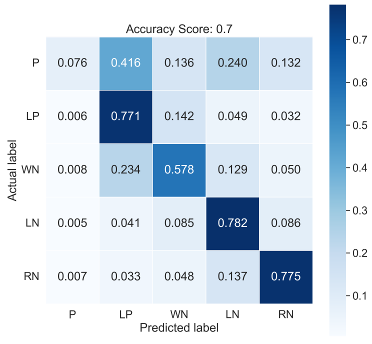

(e) RF + PUDI features

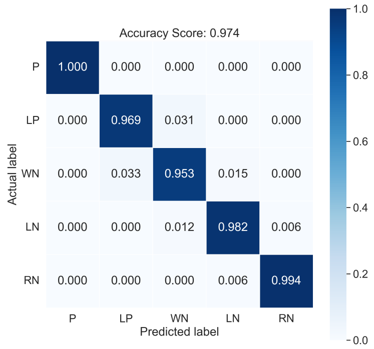

(f) RF + NeDBIT features

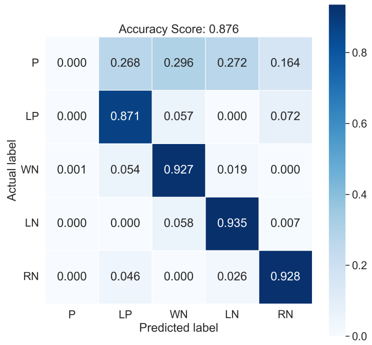

(g) SVM + TFO features

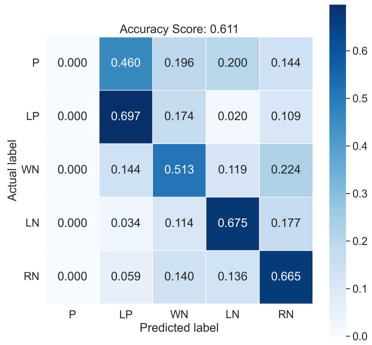

(h) SVM + PUDI features

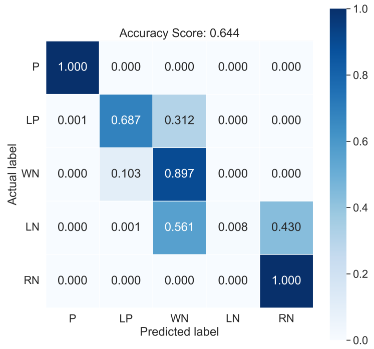

(i) SVM + NeDBIT features

Figure 1: Confusion matrices for multi-class classification on **schizophrenia** (C0036341) over the test set.

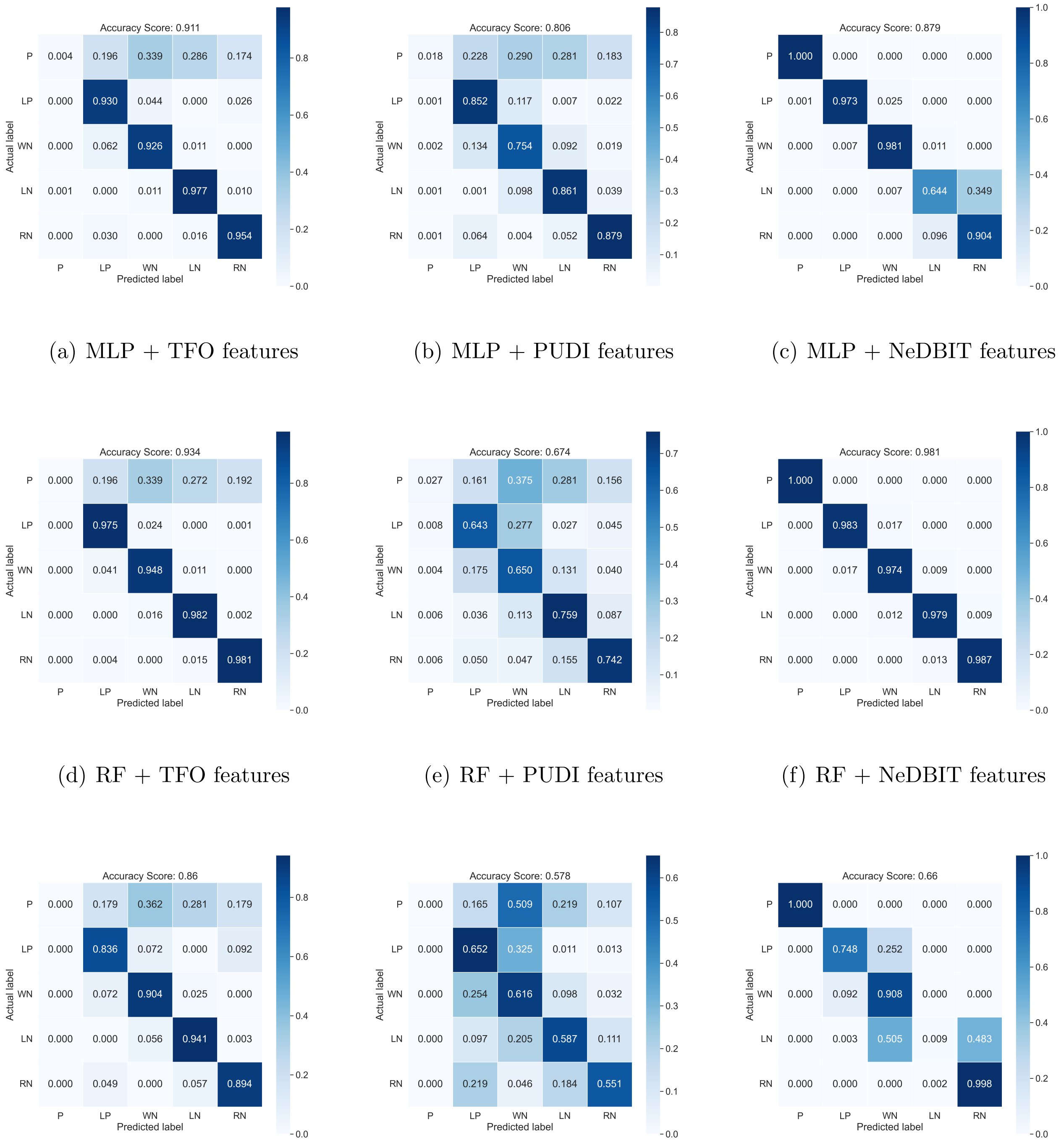

(a) MLP + TFO features (b) MLP + PUDI features (c) MLP + NeDBIT features

(d) RF + TFO features (e) RF + PUDI features (f) RF + NeDBIT features

(g) SVM + TFO features (h) SVM + PUDI features (i) SVM + NeDBIT features

Figure 2: Confusion matrices for multi-class classification on **liver cirrhosis** (C0023893) over the test set.

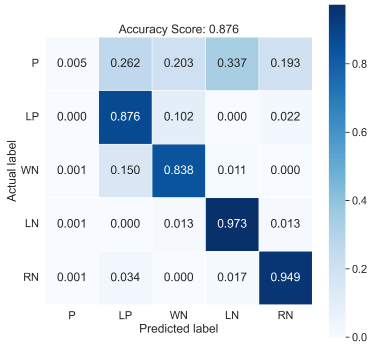

(a) MLP + TFO features

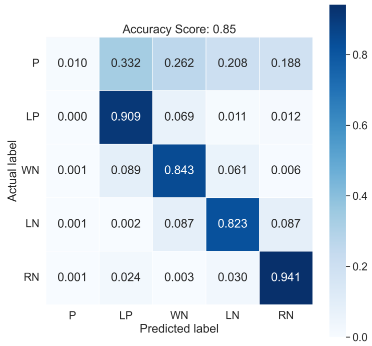

(b) MLP + PUDI features

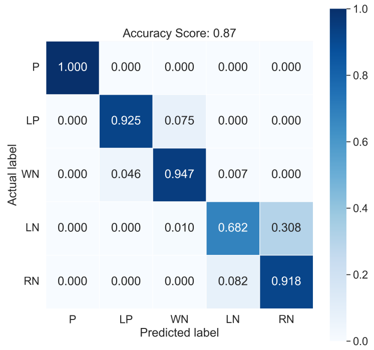

(c) MLP + NeDBIT features

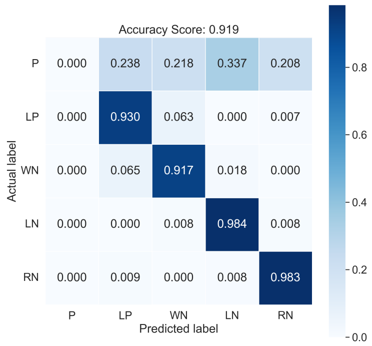

(d) RF + TFO features

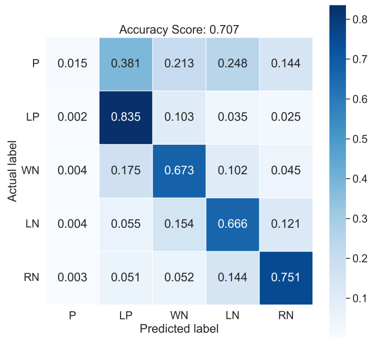

(e) RF + PUDI features

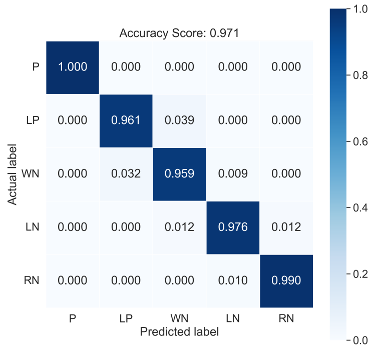

(f) RF + NeDBIT features

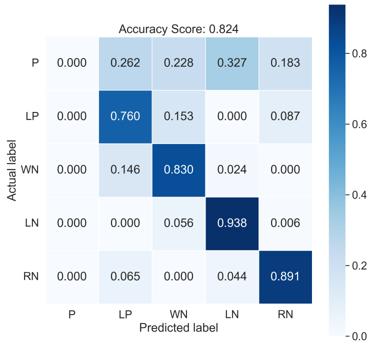

(g) SVM + TFO features

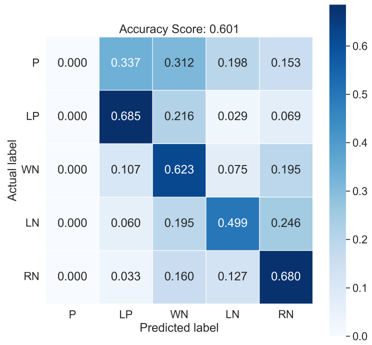

(h) SVM + PUDI features

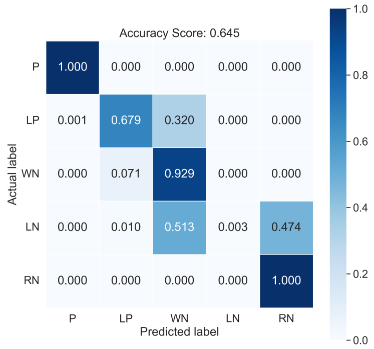

(i) SVM + NeDBIT features

Figure 3: Confusion matrices for multi-class classification on **colorectal carcinoma** (C0009402) over the test set.

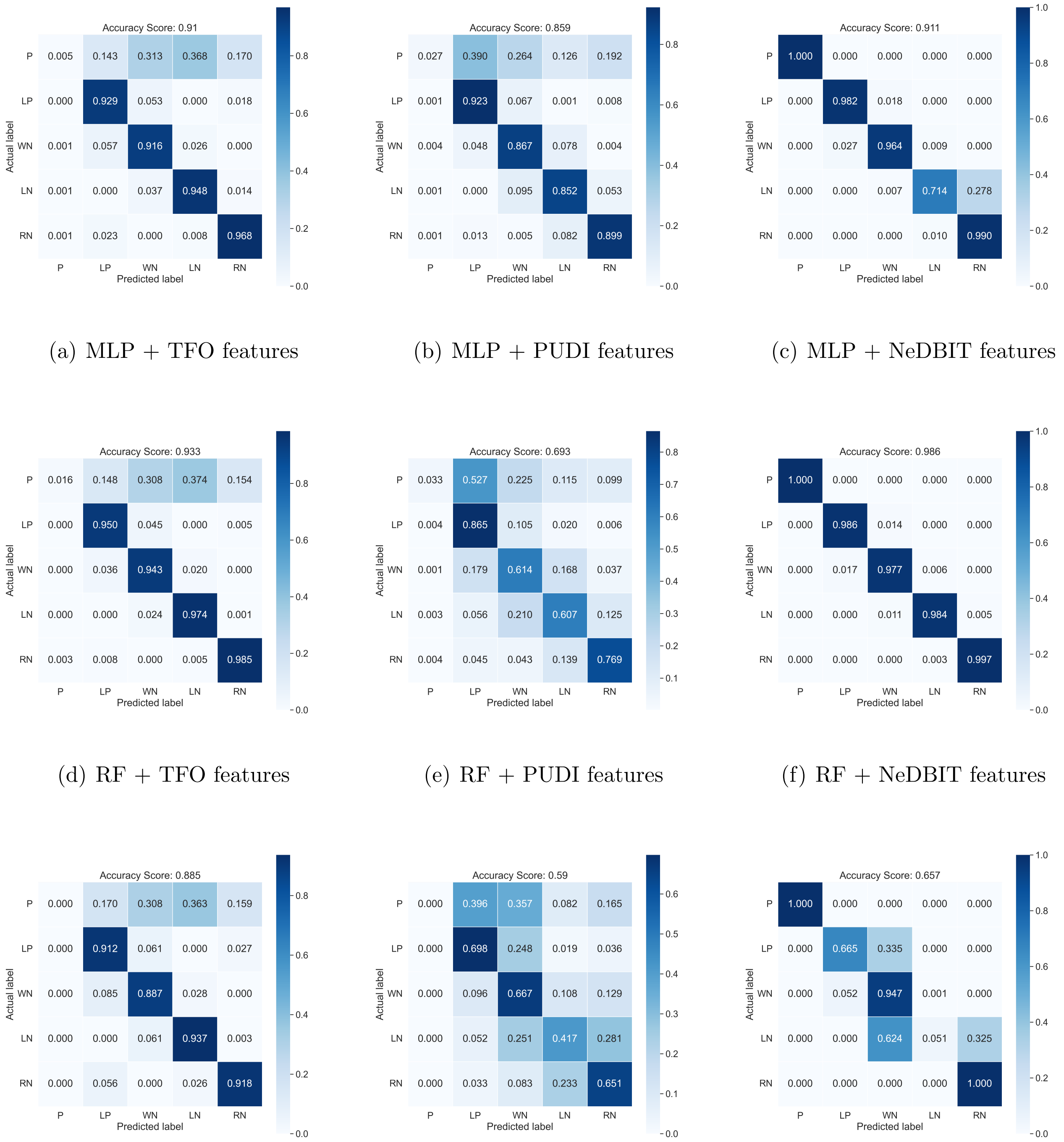

(a) MLP + TFO features (b) MLP + PUDI features (c) MLP + NeDBIT features

(d) RF + TFO features (e) RF + PUDI features (f) RF + NeDBIT features

(g) SVM + TFO features (h) SVM + PUDI features (i) SVM + NeDBIT features

Figure 4: Confusion matrices for multi-class classification on **malignant neoplasm of prostate** (C0376358) over the test set.

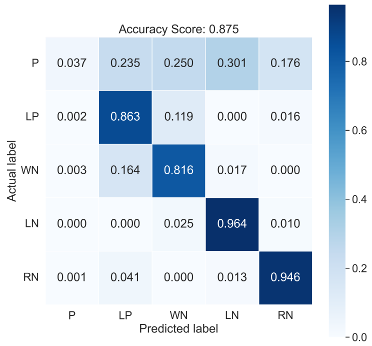

(a) MLP + TFO features

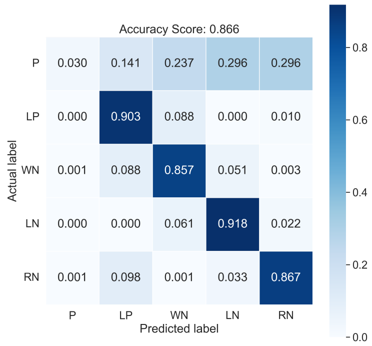

(b) MLP + PUDI features

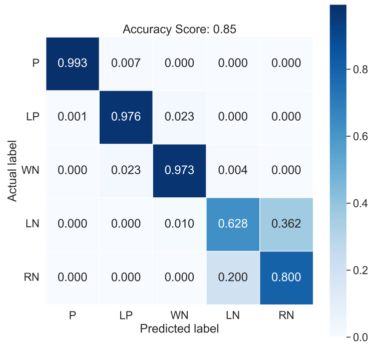

(c) MLP + NeDBIT features

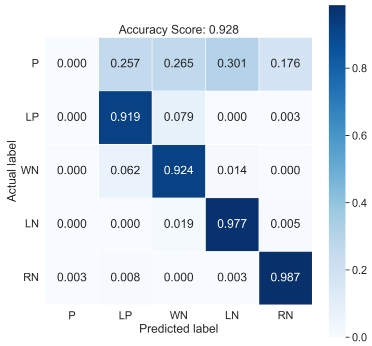

(d) RF + TFO features

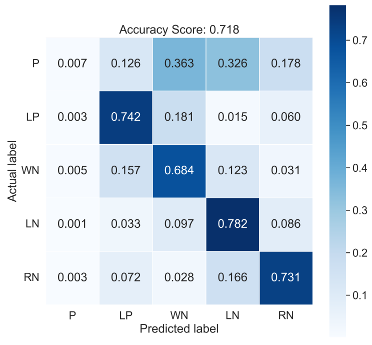

(e) RF + PUDI features

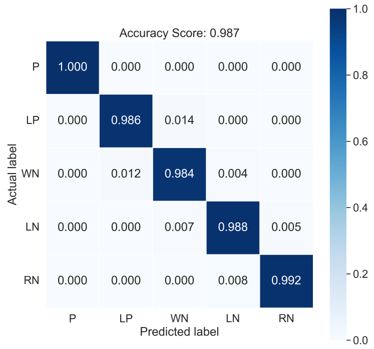

(f) RF + NeDBIT features

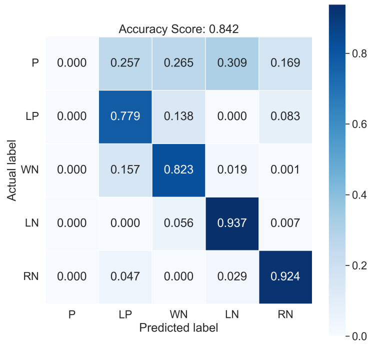

(g) SVM + TFO features

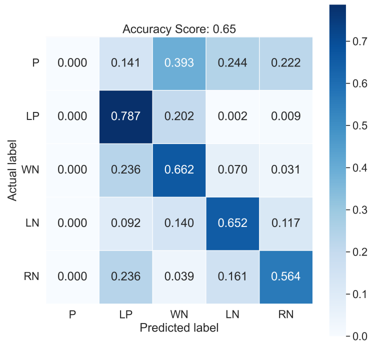

(h) SVM + PUDI features

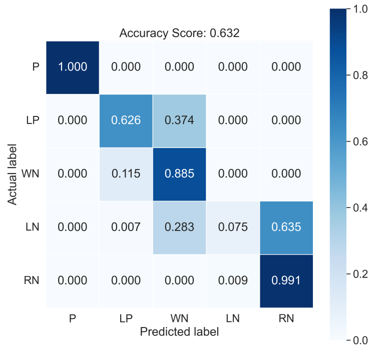

(i) SVM + NeDBIT features

Figure 5: Confusion matrices for multi-class classification on **bipolar disorder** (C0005586) over the test set.

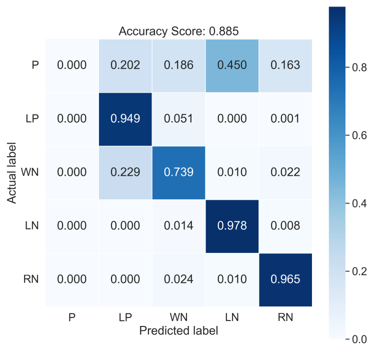

(a) MLP + TFO features

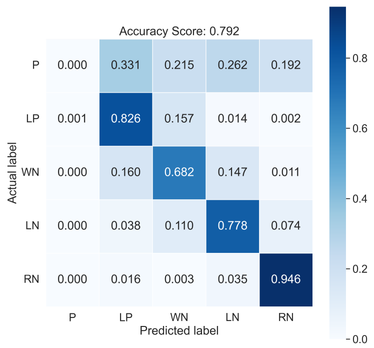

(b) MLP + PUDI features

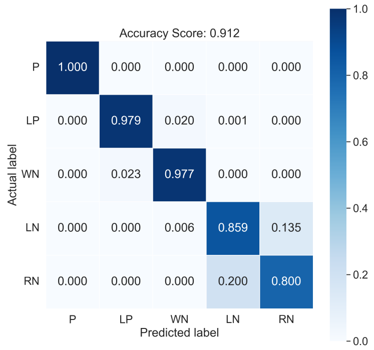

(c) MLP + NeDBIT features

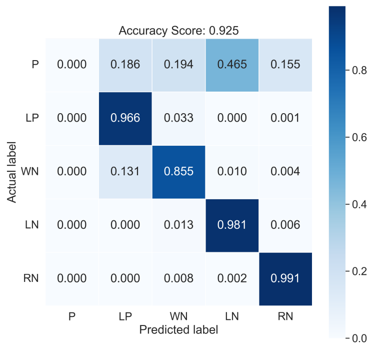

(d) RF + TFO features

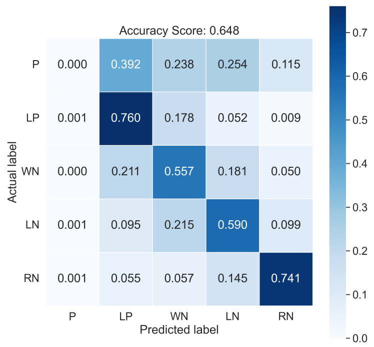

(e) RF + PUDI features

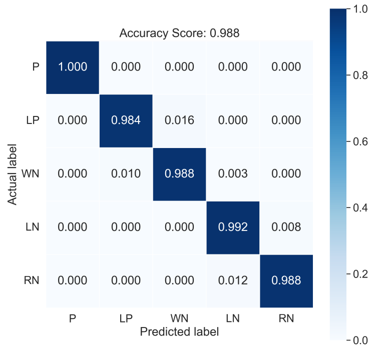

(f) RF + NeDBIT features

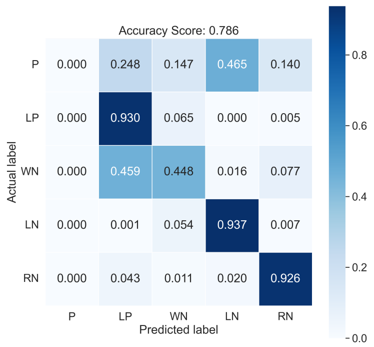

(g) SVM + TFO features

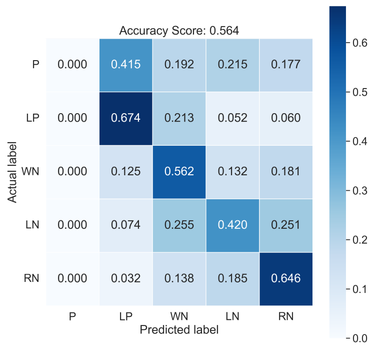

(h) SVM + PUDI features

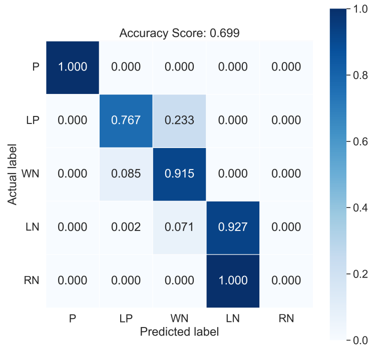

(i) SVM + NeDBIT features

Figure 6: Confusion matrices for multi-class classification on **intellectual disability** (C3714756) over the test set.

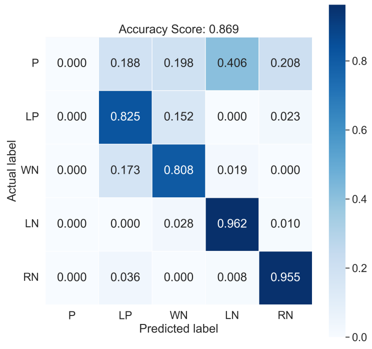

(a) MLP + TFO features

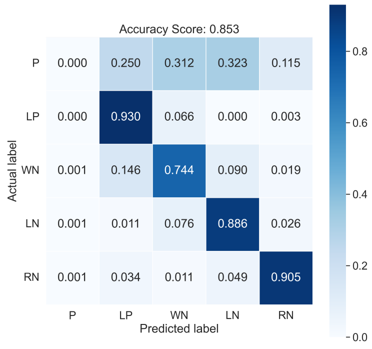

(b) MLP + PUDI features

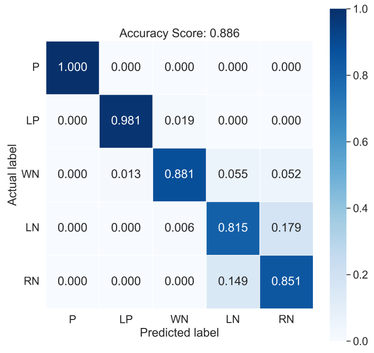

(c) MLP + NeDBIT features

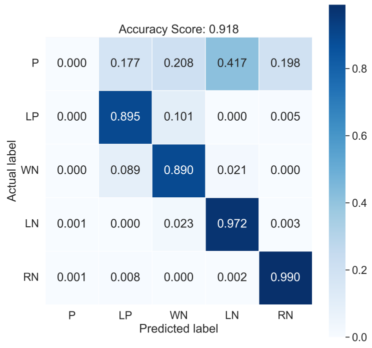

(d) RF + TFO features

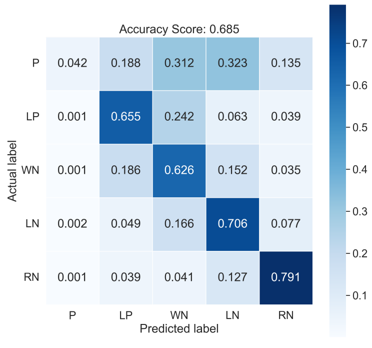

(e) RF + PUDI features

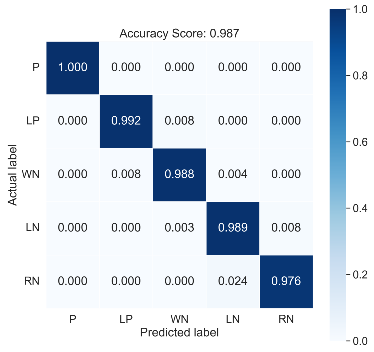

(f) RF + NeDBIT features

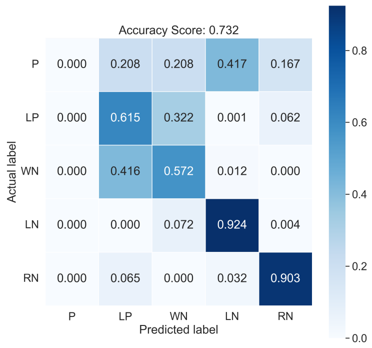

(g) SVM + TFO features

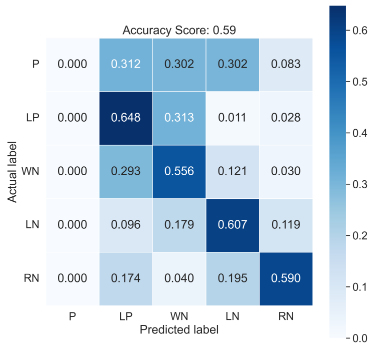

(h) SVM + PUDI features

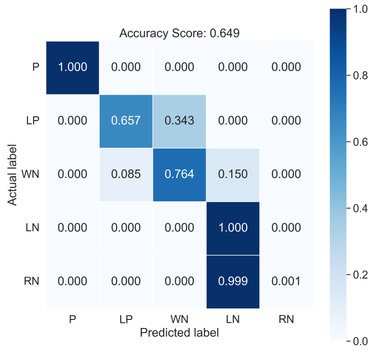

(i) SVM + NeDBIT features

Figure 7: Confusion matrices for multi-class classification on **drug-induced liver disease** (C0860207) over the test set.

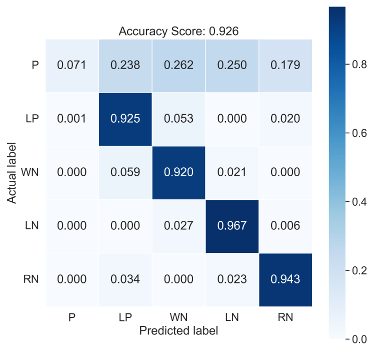

(a) MLP + TFO features

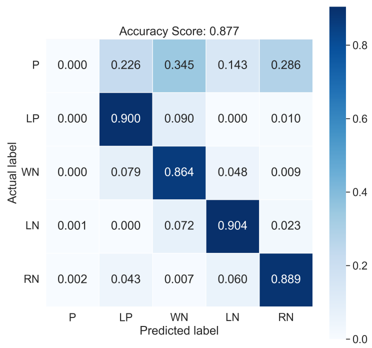

(b) MLP + PUDI features

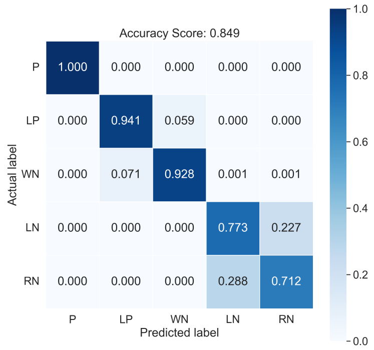

(c) MLP + NeDBIT features

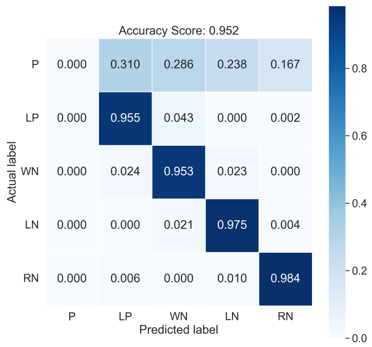

(d) RF + TFO features

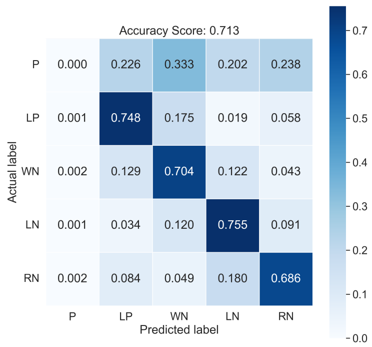

(e) RF + PUDI features

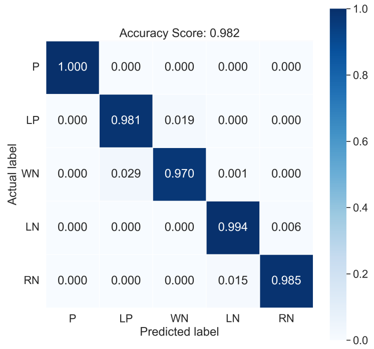

(f) RF + NeDBIT features

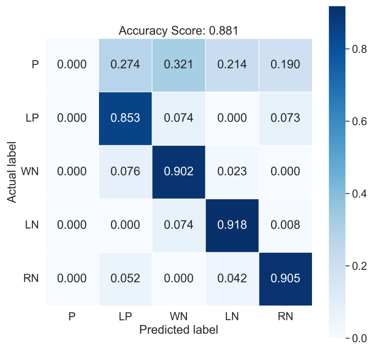

(g) SVM + TFO features

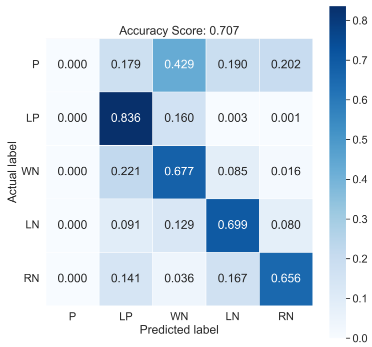

(h) SVM + PUDI features

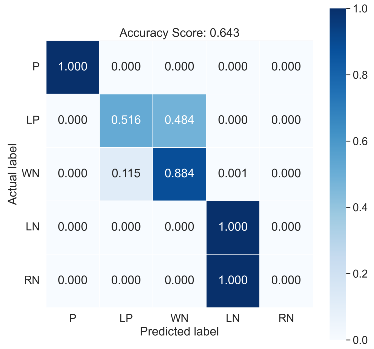

(i) SVM + NeDBIT features

Figure 8: Confusion matrices for multi-class classification on **depressive disorder** (C0011581) over the test set.

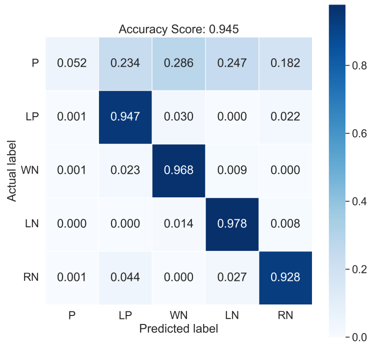

(a) MLP + TFO features

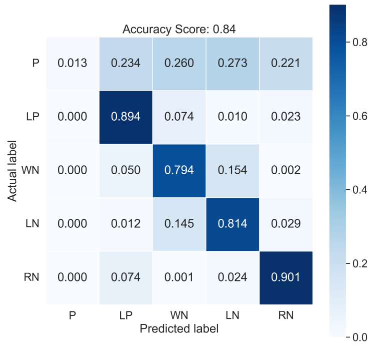

(b) MLP + PUDI features

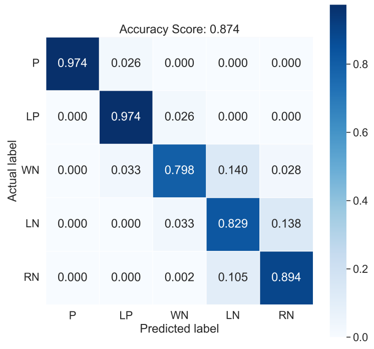

(c) MLP + NeDBIT features

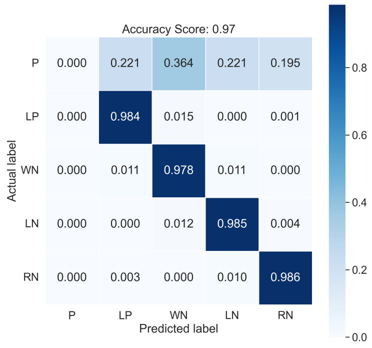

(d) RF + TFO features

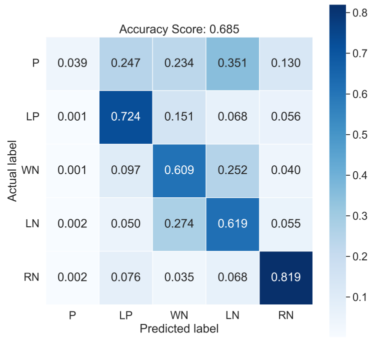

(e) RF + PUDI features

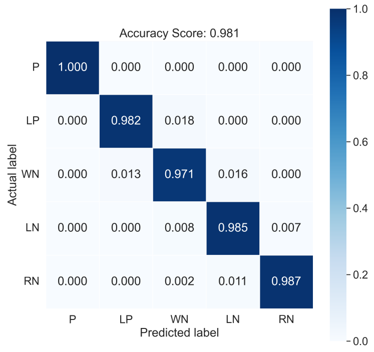

(f) RF + NeDBIT features

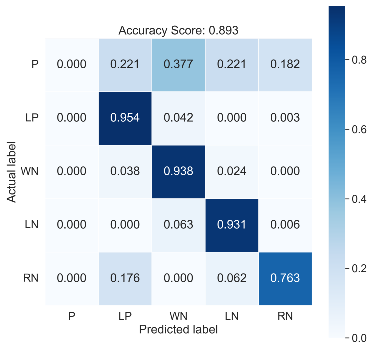

(g) SVM + TFO features

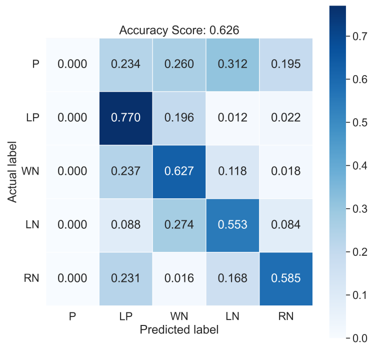

(h) SVM + PUDI features

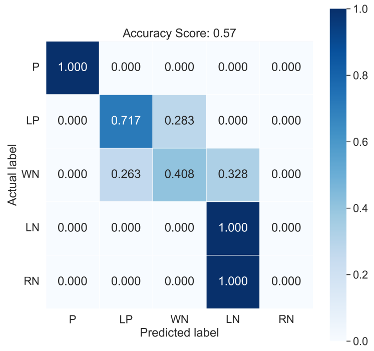

(i) SVM + NeDBIT features

Figure 9: Confusion matrices for multi-class classification on **chronic alcoholic intoxication** (C0001973) over the test set.

8 NIAPU performances in disease gene identification

In this section we present the label assignment for the 20% of seed genes which had their label removed (malignant neoplasms of breast in the main paper). Results are shown for each disease as mean with standard deviation over five runs with no overlapping masked-out gene sets. On average, 45% of the removed genes are labeled as LP. Moreover, Fig. 10 depicts the performances on the extended dataset for the disease not shown in the main paper, delivering the same insights apart for disease which represent harder tasks for all the strategies considered.

Table 4: labeling of the unlabeled seed genes by NIAPU for **schizophrenia** (C0036341).

label	% genes	# genes	GDA mean	GDA median	GDA mode
LP	44.351 ± 5.601	73.8 ± 9.311	0.369 ± 0.006	0.33 ± 0.007	0.31 ± 0.0
WN	25.602 ± 2.98	42.6 ± 4.93	0.362 ± 0.018	0.334 ± 0.021	0.312 ± 0.004
LN	21.995 ± 2.87	36.6 ± 4.775	0.361 ± 0.02	0.332 ± 0.016	0.332 ± 0.038
RN	8.052 ± 1.92	13.4 ± 3.209	0.34 ± 0.014	0.323 ± 0.021	0.308 ± 0.004

Table 5: labeling of the unlabeled seed genes by NIAPU for **liver cirrhosis** (C0023893).

label	% genes	# genes	GDA mean	GDA median	GDA mode
LP	43.229 ± 3.903	64.6 ± 6.066	0.319 ± 0.006	0.3 ± 0.0	0.3 ± 0.0
WN	25.843 ± 3.002	38.6 ± 4.393	0.308 ± 0.007	0.3 ± 0.0	0.3 ± 0.0
LN	22.895 ± 2.773	34.2 ± 4.087	0.306 ± 0.007	0.3 ± 0.0	0.3 ± 0.0
RN	8.034 ± 1.584	12.0 ± 2.345	0.3 ± 0.0	0.3 ± 0.0	0.3 ± 0.0

Table 6: labeling of the unlabeled seed genes by NIAPU for **colorectal carcinoma** (C0009402).

label	% genes	# genes	GDA mean	GDA median	GDA mode
LP	39.433 ± 3.074	53.0 ± 4.183	0.376 ± 0.022	0.331 ± 0.018	0.3 ± 0.0
WN	27.678 ± 2.854	37.2 ± 3.834	0.339 ± 0.021	0.31 ± 0.007	0.3 ± 0.0
LN	19.637 ± 4.154	26.4 ± 5.639	0.321 ± 0.013	0.3 ± 0.0	0.3 ± 0.0
RN	13.252 ± 2.99	17.8 ± 3.962	0.318 ± 0.008	0.302 ± 0.004	0.3 ± 0.0

Table 7: labeling of the unlabeled seed genes by NIAPU for **malignant neoplasm of prostate** (C0376358).

label	% genes	# genes	GDA mean	GDA median	GDA mode
LP	50.821 ± 4.635	61.6 ± 5.683	0.365 ± 0.005	0.337 ± 0.012	0.36 ± 0.055
WN	27.884 ± 4.273	33.8 ± 5.215	0.34 ± 0.009	0.314 ± 0.005	0.3 ± 0.0
LN	15.513 ± 1.984	18.8 ± 2.387	0.333 ± 0.009	0.314 ± 0.01	0.32 ± 0.045
RN	5.782 ± 3.31	7.0 ± 4.0	0.338 ± 0.026	0.327 ± 0.041	0.32 ± 0.045

Table 8: labeling of the unlabeled seed genes by NIAPU for **bipolar disorder** (C0005586).

label	% genes	# genes	GDA mean	GDA median	GDA mode
LP	43.016 ± 2.275	38.8 ± 2.049	0.354 ± 0.013	0.318 ± 0.004	0.306 ± 0.005
WN	27.053 ± 2.039	24.4 ± 1.817	0.348 ± 0.013	0.315 ± 0.005	0.306 ± 0.005
LN	22.176 ± 3.261	20.0 ± 2.915	0.35 ± 0.019	0.319 ± 0.002	0.31 ± 0.007
RN	7.756 ± 3.752	7.0 ± 3.391	0.356 ± 0.053	0.342 ± 0.061	0.338 ± 0.063

Table 9: labeling of the unlabeled seed genes by NIAPU for **intellectual disability** (C3714756).

label	% genes	# genes	GDA mean	GDA median	GDA mode
LP	48.022 ± 4.143	41.4 ± 3.647	0.368 ± 0.017	0.352 ± 0.044	0.32 ± 0.045
WN	30.853 ± 3.754	26.6 ± 3.286	0.358 ± 0.012	0.35 ± 0.046	0.32 ± 0.045
LN	16.012 ± 3.024	13.8 ± 2.588	0.369 ± 0.027	0.364 ± 0.049	0.362 ± 0.052
RN	5.114 ± 3.252	4.4 ± 2.793	0.357 ± 0.042	0.328 ± 0.041	0.3 ± 0.0

Table 10: labeling of the unlabeled seed genes by NIAPU for **drug-induced lived disease** (C0860207).

label	% genes	# genes	GDA mean	GDA median	GDA mode
LP	44.062 ± 4.044	28.2 ± 2.588	0.304 ± 0.002	0.3 ± 0.0	0.3 ± 0.0
WN	36.875 ± 3.423	23.6 ± 2.191	0.306 ± 0.003	0.3 ± 0.0	0.3 ± 0.0
LN	13.125 ± 4.765	8.4 ± 3.05	0.315 ± 0.016	0.3 ± 0.0	0.3 ± 0.0
RN	5.938 ± 2.567	3.8 ± 1.643	0.312 ± 0.027	0.3 ± 0.0	0.3 ± 0.0

Table 11: labeling of the unlabeled seed genes by NIAPU for **depressive disorder** (C0011581).

label	% genes	# genes	GDA mean	GDA median	GDA mode
LP	48.032 ± 10.381	26.8 ± 5.805	0.368 ± 0.011	0.331 ± 0.009	0.304 ± 0.005
WN	31.87 ± 7.285	17.8 ± 4.147	0.357 ± 0.017	0.328 ± 0.004	0.346 ± 0.05
LN	13.636 ± 5.214	7.6 ± 2.881	0.336 ± 0.021	0.318 ± 0.018	0.304 ± 0.005
RN	6.461 ± 2.439	3.6 ± 1.342	0.338 ± 0.036	0.326 ± 0.026	0.302 ± 0.004

Table 12: labeling of the unlabeled seed genes by NIAPU for **chronic alcoholic intoxication** (C0001973).

label	% genes	# genes	GDA mean	GDA median	GDA mode
LP	40.0 ± 9.566	20.4 ± 4.879	0.356 ± 0.017	0.324 ± 0.005	0.308 ± 0.004
WN	28.627 ± 6.138	14.6 ± 3.13	0.36 ± 0.03	0.322 ± 0.014	0.308 ± 0.004
LN	20.784 ± 6.877	10.6 ± 3.507	0.337 ± 0.026	0.316 ± 0.008	0.312 ± 0.011
RN	10.588 ± 4.514	5.4 ± 2.302	0.328 ± 0.022	0.311 ± 0.002	0.306 ± 0.005

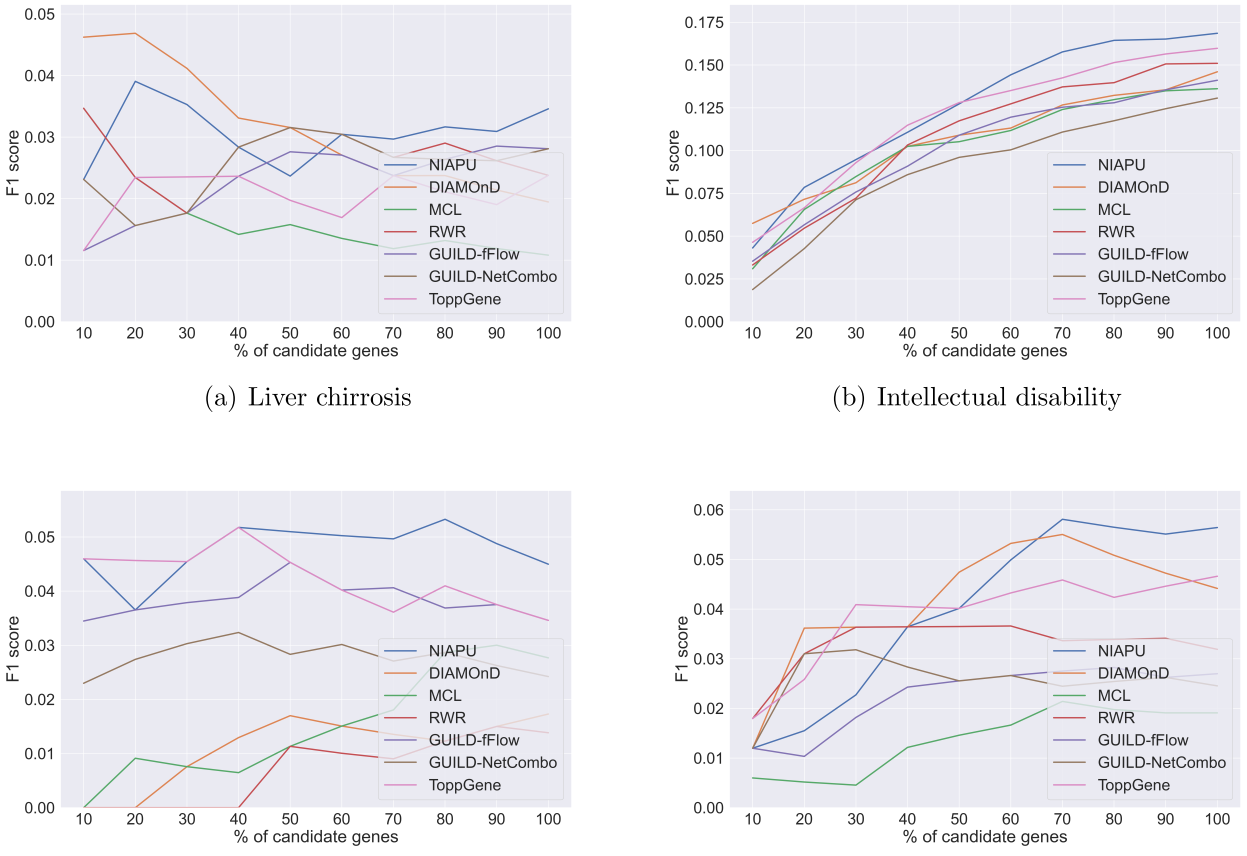

(a) Liver chirrosis

(b) Intellectual disability

(c) Drug-induced liver disease

(d) Chronic alcoholic intoxication

Figure 10: Gene discovery performances in terms of F1 score. Results are reported for four diseases at increasing number of candidate genes considered as percentage of the total number of associated genes in the extended dataset, which is different for each disease.

9 Description of the disease gene discovery tools

The method implemented in DIAMOnD focuses on the connectivity properties of the network. More specifically, in DIAMOnD the concept of connectivity significance (CS) is defined as the cumulative probability of a given protein to have more connections to seed proteins than expected. The approaches relies on the observation that CS is the best predictive measure for a disease associated protein. Through the calculation and ranking of the CS of all proteins connected to known seed proteins, it is possible to evaluate which protein has more connections than expected to seed proteins. The protein showing the lowest p-value (i.e., the greatest significance) is considered a most likely putative disease protein, and it is added to the seed protein set for another iteration to discover the next most likely putative protein.

MCL is based on simulation of stochastic flow in graphs. Based on such principle, clusters are created, and the presence of known disease genes in a cluster make the other elements putative disease genes. Quite similarly, RWR follows the guilt-by-association strategy, exploring the PPI network vicinity of known disease genes based on the premise that nodes related to similar functions tend to lie close to each other.

The method implemented in GUILD is based on the hypothesis that the interconnections among disease genes in the interactome are captured by taking into account the "relevance" of the paths connecting the disease genes, and exploits topology-based ranking algorithms such as NetShort, NetZcore and NetScore, combined to provide the consensus method NetCombo, among others.

Finally, ToppGene exploits a fuzzy-based similarity measure to compute the similarity between any two genes based on semantic annotations as well as extended versions of the PageRank and HITS algorithms, and the K-Step Markov method calculated over the interactome topology.

References

[1] Guido Van Rossum and Fred L. Drake. *Python 3 Reference Manual*. Scotts Valley, CA: CreateSpace, 2009. ISBN: 1441412697.

[2] Aric Hagberg, Pieter Swart, and Daniel S Chult. *Exploring network structure, dynamics, and function using NetworkX*. Tech. rep. Los Alamos National Lab.(LANL), Los Alamos, NM (United States), 2008.

[3] Jianzhen Xu and Yongjin Li. "Discovering disease-genes by topological features in human protein–protein interaction network". In: *Bioinformatics* 22.22 (Sept. 2006), pp. 2800–2805. DOI: 10.1093/bioinformatics/btl467. URL: https://doi.org/10.1093/bioinformatics/btl467.

[4] Nadezhda T. Doncheva, Tim Kacprowski, and Mario Albrecht. "Recent approaches to the prioritization of candidate disease genes". In: *WIREs Systems Biology and Medicine* 4.5 (June 2012), pp. 429–442. DOI: 10.1002/wsbm.1177. URL: https://doi.org/10.1002/wsbm.1177.

[5] Seth Carbon and Chris Mungall. *Gene Ontology Data Archive*. Version 2022-03-22. Zenodo, July 2018. DOI: 10.5281/zenodo.6399963. URL: https://doi.org/10.5281/zenodo.6399963.

[6] S. D. Ghiassian, J. Menche, and A. L. Barabási. "A DIseAse MOdule Detection (DIAMOnD) algorithm derived from a systematic analysis of connectivity patterns of disease proteins in the human interactome". In: *PLoS Comput Biol* 11.4 (Apr. 2015), e1004120.

[7] Diederik P Kingma and Jimmy Ba. "Adam: A method for stochastic optimization". In: *arXiv preprint arXiv:1412.6980* (2014).

[8] Martín Abadi et al. *TensorFlow: Large-Scale Machine Learning on Heterogeneous Systems*. Software available from tensorflow.org. 2015. URL: https://www.tensorflow.org/.

[9] François Chollet et al. *Keras*. https://keras.io. 2015.

[10] F. Pedregosa et al. "Scikit-learn: Machine Learning in Python". In: *Journal of Machine Learning Research* 12 (2011), pp. 2825–2830.
